# Leukocyte-specific protein 1 is associated with the stage and tumor immune infiltration of cervical cancer

**DOI:** 10.1038/s41598-025-91066-0

**Published:** 2025-03-04

**Authors:** Dianqin Xu, Xinzhu Zhou, Shaoju Min, Yu Zhang, Xiaoyu Zhu, Kun Qiao, Luhong Xie, Ji Ren, Yameng Liu, Ziwen Xiao, Yujie Tan

**Affiliations:** 1 Centre for Clinical Laboratories, The Affiliated Hospital of Guizhou Medical University, Guiyang, 550004 Guizhou China; 2 School of Clinical Laboratory Science, Guizhou Medical University, Guiyang, 550004 Guizhou China; 3 The Affiliated Hospital of Guizhou Medical University, Hypertension Department, Guiyang, 550004 Guizhou China; 4 Department of Obstetrics and Gynaecology, The Affiliated Hospital of Guizhou Medical University, Guiyang, 550004 Guizhou China; 5 Guizhou Hospital of The First Affiliated Hospital, SUN Yat-Sen University, Guiyang, 550004 Guizhou China

**Keywords:** Cervical cancer, HPV16, Leucocyte-specific protein 1 (LSP1), Tumour infiltrating lymphocytes (TILs), Tumour microenvironment (TME), Cancer, Computational biology and bioinformatics, Immunology, Biomarkers, Oncology, Pathogenesis

## Abstract

Cervical cancer (CC) is a leading cause of cancer-related mortality among women and is strongly associated with persistent infection by high-risk human papillomavirus (HR-HPV), particularly the HPV16 subtype. Existing detection methods have limitations in meeting clinical requirements. This study aims to identify biomarkers that can aid in the staging and prognosis of cervical cancer. Cervical epithelial exfoliated cell samples were collected from three groups: HPV16-negative normal cervix, HPV16-positive normal cervix, and HPV16-positive cervical cancer. Differential expression proteins (DEPs) were identified using TMT-LC–MS/MS technology, and their associations with tumor-infiltrating lymphocytes (TILs) and immune regulatory molecules were analyzed. Leukocyte-specific protein 1 (LSP1), an intracellular F-actin-binding protein predominantly expressed in macrophages, neutrophils, B cells, and T cells, was identified as a potential biomarker. The expression levels of LSP1 were evaluated and validated using the Human Protein Atlas (HPA) database, immunohistochemistry (IHC), Western blotting (WB), and real-time quantitative PCR (RT-qPCR). Multiplex fluorescence immunohistochemistry (mIHC) was employed to assess the co-localization of LSP1 with CD4^+^ and CD8^+^ T cells. Results indicated that both protein and mRNA levels of LSP1 were significantly elevated in cervical cancer tissues compared to adjacent non-tumor tissues. Notably, LSP1 expression was higher in early-stage cervical cancer (Stage IB) than in advanced-stage disease (Stage IIIC). Furthermore, LSP1 was predominantly localized in CD4^+^ and CD8^+^ T cells with low TIM-3 expression. Analysis of public databases (GEPIA, TIMER2.0, and TISIDB) revealed that higher LSP1 mRNA levels correlated with better patient outcomes. LSP1 expression was positively associated with the abundance of major TILs and immune regulatory molecules, particularly activated B cells, CD8^+^ T cells, and CD4^+^ T cells, while negatively correlated with M2 macrophages and myeloid-derived suppressor cells. These findings indicate that the expression levels of LSP1 in cervical tissues are correlated with cancer staging and patient prognosis, potentially reflecting both tumor immune infiltration and T-cell exhaustion within the tumor microenvironment (TME).

## Introduction

Cervical cancer, with an umbrella designation “cervical squamous cell carcinoma and endocervical adenocarcinoma (CESC)”, is a major gynaecologic tumour associated with persistent infection of high-risk human papillomaviruses (HR-HPVs)^1^. The prognosis of cervical cancer is usually poor due to the lack of tools for early detection and treatment with sufficient specificity and sensitivity. Carcinogenesis of cervical epithelial cells is often preceded by cervical intraepithelial neoplasia (CIN), a continuum of precancerous lesions characterised by abnormal growth of cells outlining the cervix^2^. HR-HPVs are also responsible for oropharyngeal cancers and anogenital cancers other than cervical cancer^3^. Among 15 confirmed carcinogenic HPV viruses, geographical variance notwithstanding, HPV16, HPV18, HPV31, HPV33, HPV52, and HPV58 subtypes are the most prevalent^1,3^. HPV16 alone accounts for 60.5% of all cervical cancer cases, followed by HPV18^4^. Our previous study showed that 94.90% of cervical cancer patients in Guizhou province, China, were positive for HR-HPV subtypes HPV16, HPV52, and HPV58^5^. As the most predominant HR-HPVs, HPV16 and HPV18 pose a preferential risk for squamous cell carcinoma and cervical adenocarcinoma, respectively^1,6–8^. Low-risk HPV mainly includes HPV6, HPV11, and HPV43 subtypes, which chiefly cause vulvar condyloma lesions^9^. Ample evidence has confirmed that the overexpression of E6/E7 oncoprotein is the hallmark of HR-HPV virulence leading to CINII/ CINIII and cervical cancer^4^. The long incubation period from virus invasion to malignant transformation, approximately 5–10 years^10^, implies that the initial episode of HR-HPV infection might provide the best opportunity for cervical cancer prevention and early diagnosis and treatment. However, the expressional and functional profile of key proteins associated with HPV16 infection and disease evolvement is far from clear^11^.

Following HR-HPV infection, the establishment of an immunosuppressive environment facilitates a second hit to foster cancer development. The composition and infiltration of immune cells (TILs) in the tumour microenvironment (TME) are crucial players in tumour progression and response to immunotherapy^12^. Insufficient or impaired HPV-specific T-cell immunity is regarded as the main factor in determining virus clearance or cancer development^13^. Immunotherapy strategies against cervical cancer, such as induction or reactivation of T-cells targeting E6/E7 antigens or reversion of effector T-cell suppression are of great interest^14,15^. Another attractive target is the elevated immune checkpoint control that promotes immune escape, such as PD-L1 overexpression which is found in up to 80% of cervical cancer^16^. However, one of the major obstacles to the efficacy of cell-based cancer immunotherapy is the insufficient recruitment of T-cells inside the tumour^17^.

Leukocyte-specific protein (LSP1) is an intracellular F-actin-binding protein, mainly expressed by haematopoietic cells and endothelium cells^18^. There is some evidence to place the role of LSP1 in solid tumours, either driving or inhibiting cancer progression. LSP1 is one of the five genes with single nucleotide polymorphisms associated with breast cancer susceptibility^19,20^. In a copy number variation study of human hepatocellular carcinoma tissues, LSP1 is identified as the most frequent gene that manifests deletion and amplifications^21^. A study of RNA sequencing of bladder cancer cells identified a correlation between LSP1 downregulation and pictilisib resistance^22^. LSP1 plays a negative regulatory role in cell proliferation and migration of hepatocellular carcinoma, as shown in cell lines and mice^23,24^. Meanwhile, there are studies contradicting the concept of LSP1 as a tumour suppressor. In a study of glioblastoma, high levels of LSP1 are accompanied by elevated immune infiltration that favours the immunosuppressive TME and poor response to radio/chemotherapy, suggesting that LSP1 might serve as a progressive malignancy marker^25^. It has been reported that a lack of LSP1 promoted T-cell infiltration in a mouse model of melanoma, while enhanced LSP1 levels impeded the chemotactic migration of CD8^+^ T cells via CXCL9/CXCL10 axis^26^. Little is known about whether the actin-binding and immunomodulation capacity of LSP1 affects HPV virus clearance.

In the present study, by analysing the data we collected for the previous quantitative proteomics study^27^, we identified early changes in LSP1 upon HPV16 infection. We focused on the specific associations between the presence of LSP1 and the infiltration of lymphocyte subtypes that are relevant to immunosuppression and anti-tumour activity in cervical cancer.

## Results

### Differentially expressed proteins (DEPs) are identified among the comparison groups

**Fig. 1:**
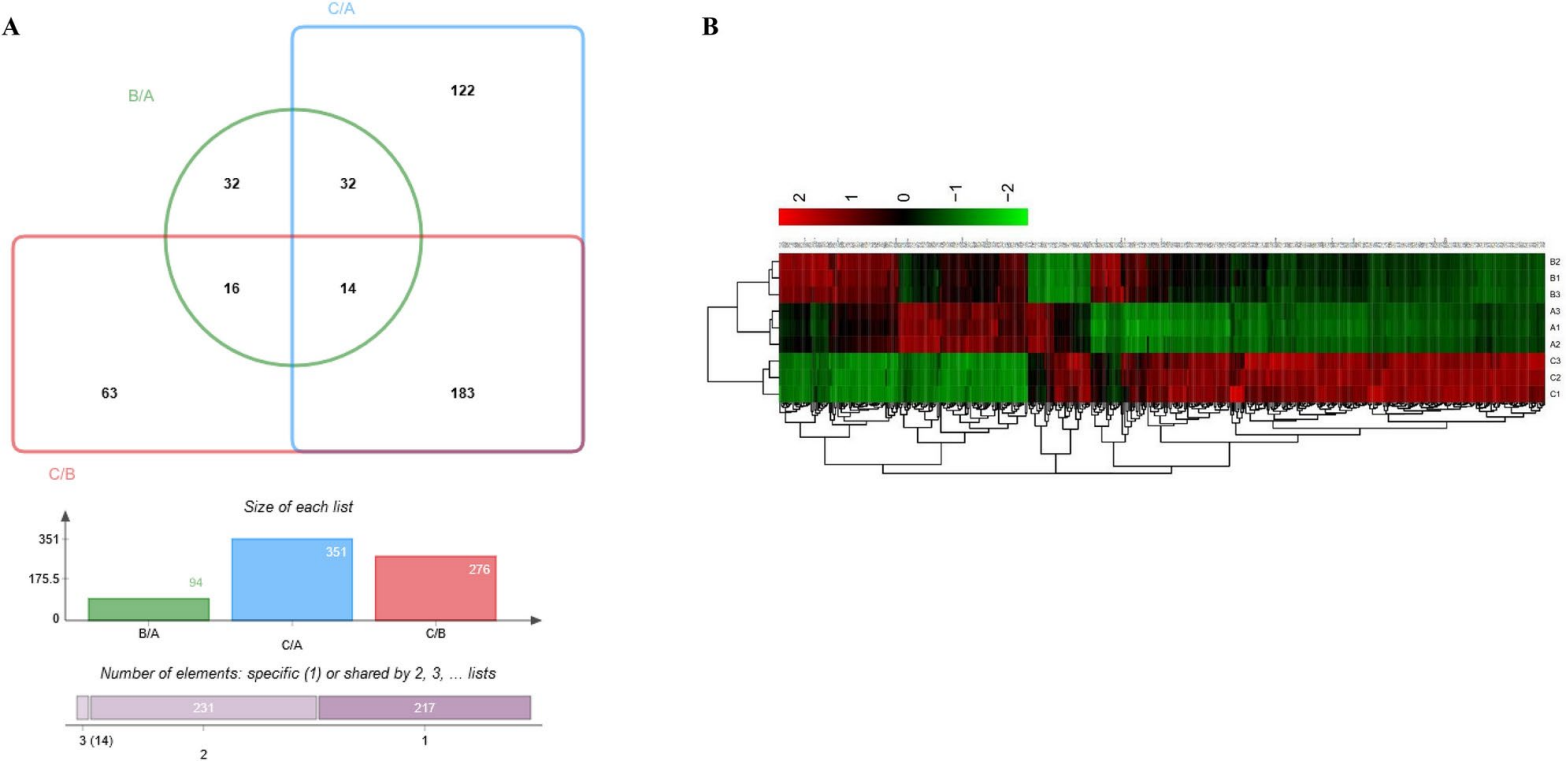
Venn diagram showing the overlap of specific and common differential expressed proteins (DEPs) identified among the comparison groups (**A**) and the expression pattern clustering heat map of DEPs (**B**) in three biological replicates of HPV16 negative normal (A1, A2 and A3), HPV16 positive normal (B1, B2 and B3), and HPV16 positive cervical cancer (C1, C2 and C3). (**A**) Green oval, blue rectangle and red rectangle represent numbers of DEPs in group B, group A and group C, respectively. Numbers in the intersections indicate the numbers of common DEPs between the groups and in the outside part are the numbers of unique DEPs in each group. (**B**) The small grids represent each DEP. The higher the expression, the darker the colour (red for up-regulation, green for down-regulation). Each row shows the relative expression level of individual DEPs in each group, and each column shows the expression differences of each DEP among different groups. The lower and the left dendrogram represent the cluster analysis results of data from each group and across the groups, respectively. The values beside the colour key indicate fold changes.

A Venn diagram was generated to present the unique and common DEPs in comparisons between groups **(**Fig. 1A**)**. 94 DEPs were identified in the HPV16-positive cervical normal group (group B) compared with the HPV-negative cervical normal group (group A). Of them, 65 DEPs were upregulated, whereas 29 were downregulated. Compared with the HPV16-positive cervical normal group (group B), 276 DEPs were identified in the HPV16-positive cervical cancer group (group C), of which 157 DEPs were upregulated and 119 downregulated. Compared with the HPV-negative cervical normal group (group A), 351 DEPs were identified in the HPV16-positive cervical cancer group (group C). Of them, 247 DEPs were upregulated and 104 were downregulated. The expression pattern clustering heatmap (Fig. 1B) exhibited distinct protein expression patterns featuring HPV16 positivity or cervical carcinogenesis. A good reproducibility was observed within the replicates of each group.

### LSP1 is associated with HPV16 infection, tumour purity and immune infiltration

Using TIMER2.0 algorithms to analyse the correlation between DEPs and immune infiltrating cells in the three comparison groups, we identified DEPs that had significant correlations with tumour purity and immune infiltrating cells (Table 1). Among them, leukocyte-specific protein 1 (LSP1) was the only one whose abundance was significantly associated with HPV16 infection (group B *vs* A, FC = 0.49), but not with cancer status (group C *vs* B, FC = 1.03). Meanwhile, LSP1 was negatively correlated with tumour purity, which suggested that it was primarily expressed in infiltrating immune cells, not tumour cells. LSP1 exhibited a negative correlation with tumor purity but demonstrated a positive correlation with the infiltration levels of various immune cells, including B cells, CD8^+^ T cells, CD4^+^ T cells, neutrophils, and dendritic cells. These findings suggest that LSP1 is likely to be predominantly expressed in infiltrating immune cells rather than in tumor cells themselves. LSP1 is known to be a cytoplasmic protein expressed by immune cells and plays important immune regulatory roles^28^. The results suggested that the status of LSP1 might reflect the early immune environment stirred by HPV16 infection.

**Table 1:**
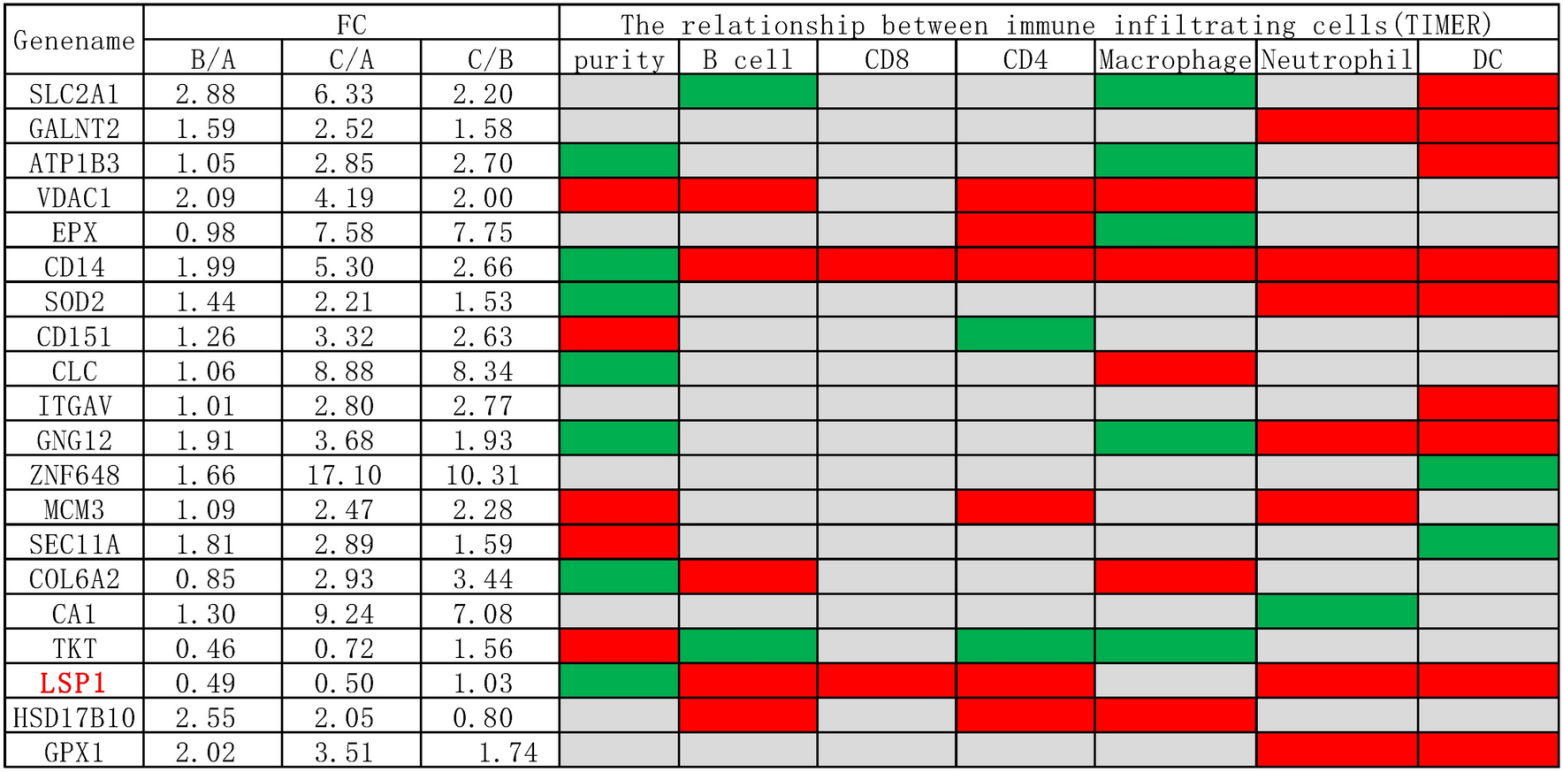
The functional heatmap table showing the fold changes (FC) of some differentially expressed proteins (DEPs) between comparison groups and their correlations with tumour purity and the key immune infiltrates in cervical cancer, estimated by TIMER2.0 algorithms. A statistically significant positive association is marked in red, while green colour indicates a statistically negative association and grey denotes a non-significant result. LSP1 is highlighted in red font.

### LSP1 expression in cervical cancer tissue is associated with non-exhausted CD4^+^ and CD8^+^ T cells

**Fig. 2:**
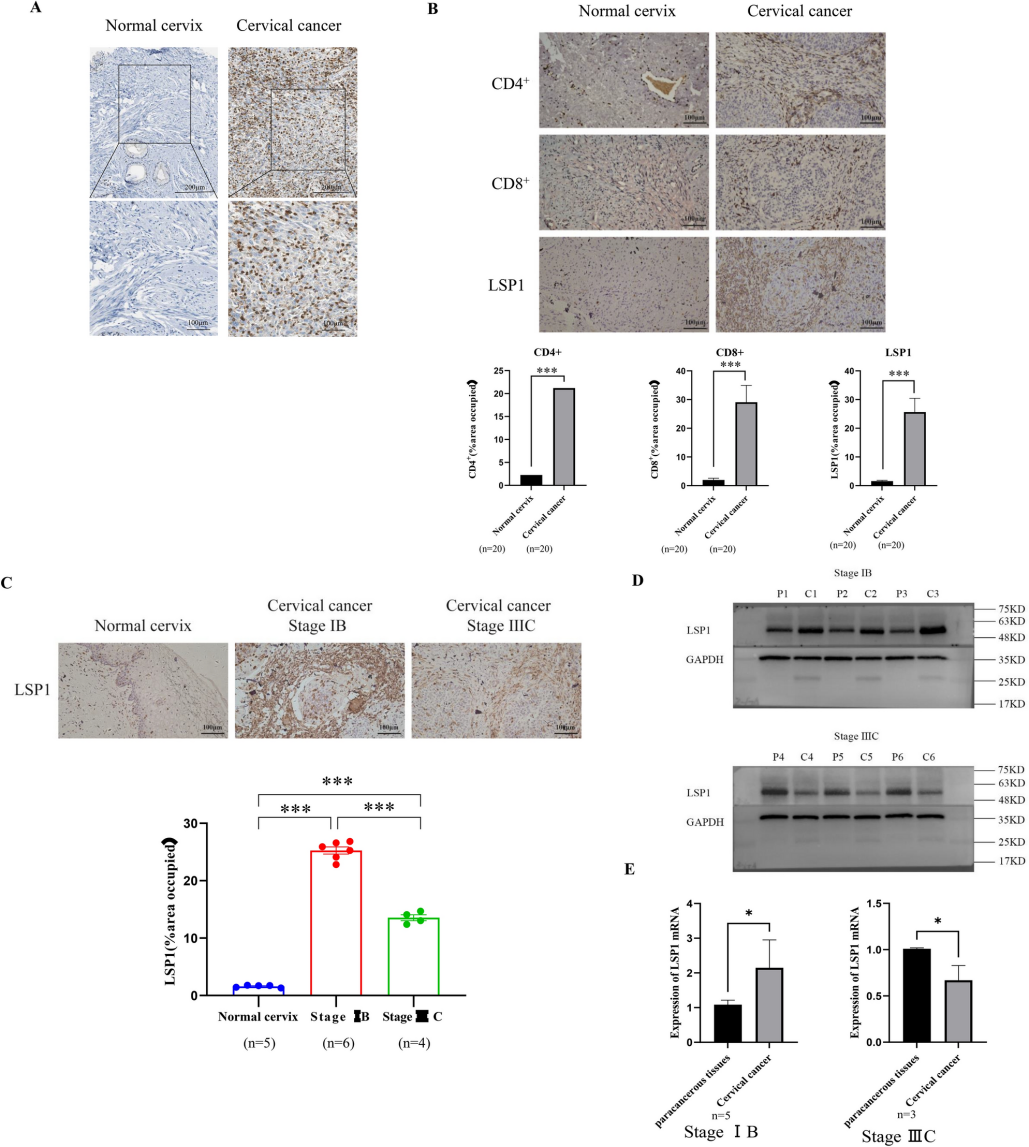
(**A**) Representative images of immunohistochemical (IHC) staining of LSP1 in normal cervix and cervical cancer tissues obtained from the Human Protein Atlas (HPA) database. (**B**) The IHC staining of LSP1, CD4^+^ and CD8^+^ T cell markers in HPV16-negative normal and HPV16-positive cervical cancer cervix tissues. (**C**) IHC analysis of LSP1 expression in normal, Stage IB cervical cancer, and Stage IIIC cervical cancer tissues.(**D**) Western blot analysis of LSP1 protein expression levels in cervical cancer and adjacent non-tumor tissues. (**E**) RT-qPCR analysis of LSP1 mRNA expression levels in cervical cancer and adjacent non-tumor tissues.

**Fig. 3:**
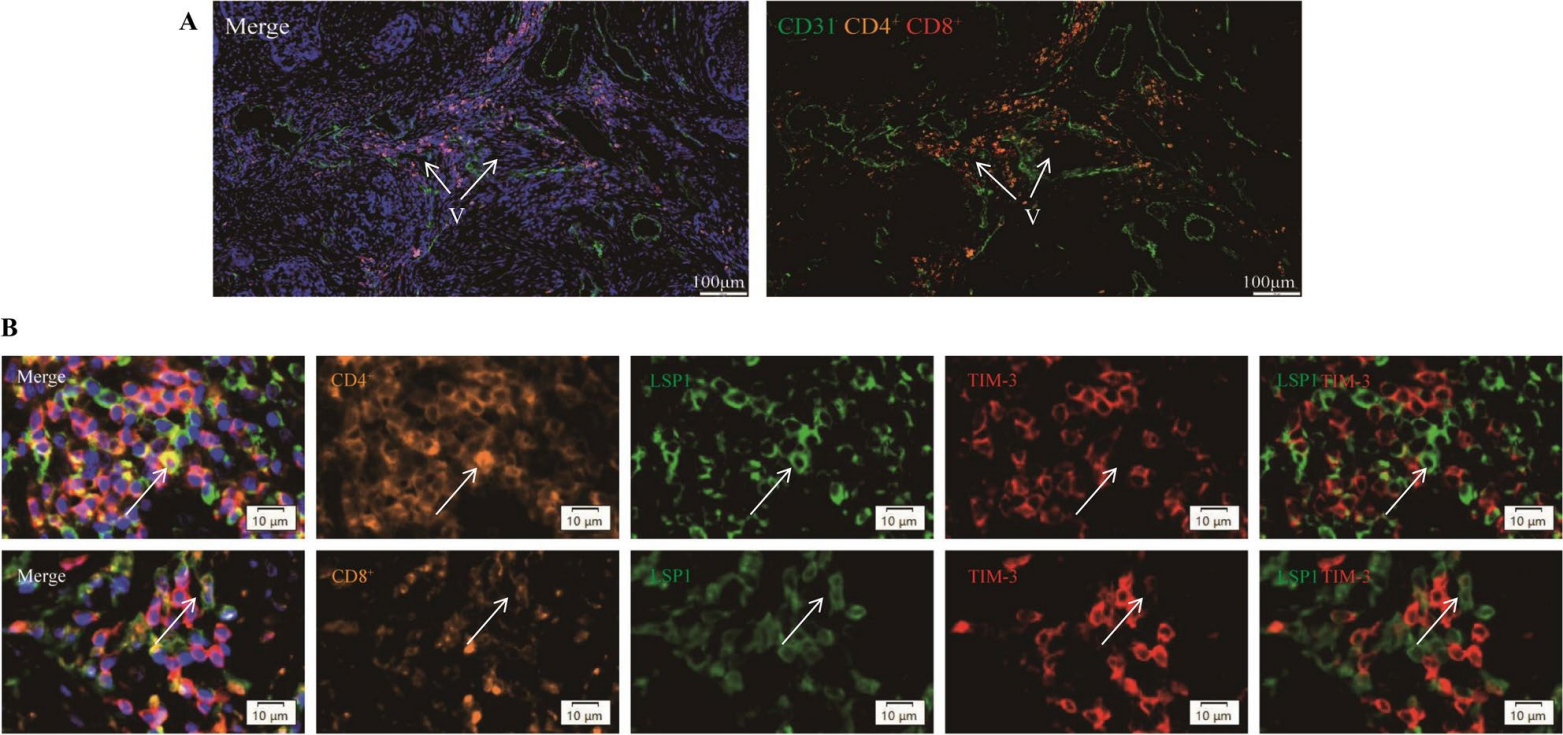
(**A**) Representative mIHC staining images showing the distribution of CD8^+^ (red) and CD4^+^ (orange) T cells around microvascular vessels. CD31 (green) serves as the marker of microvascular vessels (V, indicated by white arrows). (**B**): Representative mIHC staining images showing the co-staining of LSP1 (green) with TIM-3-expressing CD4^+^ (upper panel) and CD8^+^ (lower panel) T cells in cervical cancer tissue sections. The nucleus was stained blue by DAPI (blue).

The levels of LSP1 expression in cervical cancer tissues were next evaluated by referring to the immunohistochemical analysis of the Human Protein Atlas (HPA) database (https://www.proteinatlas.org). Surprisingly, in contrast to the proteomics data, LSP1 exhibited higher expression levels in cervical cancer tissues compared to normal cervical tissues. In addition, LSP1 was found to be distributed outside the cervical cancer cells (Fig. 2A), in cells existing in the tumour microenvironment (TME). This may explain the discrepancy between the proteomics results obtained from exfoliated cervical cells (Table 1 & Fig. 1**)** and the local protein expression in cervical tissues. Given the propensity of LSP1 expression by immune cells, we went on to verify the correlation between LSP1 expression and immune cell infiltration, using IHC and mIHC staining performed on the pathological cervical sections. IHC results revealed that LSP1, CD4^+^, and CD8^+^ T cells were highly expressed in cervical cancer tissues, in comparison with the normal cervix (Fig. 2B).Based on the FIGO staging criteria, immunohistochemistry (IHC) analysis was performed on early-stage (Stage IB) and advanced-stage (Stage IIIC) cervical cancer tissues. The results demonstrated that LSP1 expression was significantly elevated in early-stage cervical cancer (Stage IB) but markedly decreased in advanced-stage cervical cancer (Stage IIIC) (Fig. 2C). To further investigate this observation, we conducted Western blot and RT-qPCR analyses to evaluate the protein and mRNA expression levels of LSP1 in both early-stage and advanced-stage cervical cancer tissues. Consistent with the IHC findings, LSP1 expression was higher in early-stage cervical cancer (Stage IB) compared to adjacent non-tumor tissues, while it was lower in advanced-stage cervical cancer (Stage IIIC) (Fig. 2D and 2E). These findings strongly suggest that LSP1 expression is associated with the clinical stage of cervical cancer. Using mIHC staining of the cancer sections, we identified CD4^+^ and CD8^+^ T cells around microvessels (Fig. 3A). The results corroborated the explanation for the absence of LSP1 in exfoliated cells. T-cell immunoglobulin and mucin domain 3 (TIM-3) is a transmembrane protein expressed on T cells and other lymphocyte subtypes. High expression of TIM-3^+^ CD4^+^ Th1 cells and TIM-3^+^ CD8^+^ T cells plays tumour-promoting roles in HPV-positive cervical cancer^29^. Elevated TIM-3 expression in TME marks the exhaustion of CD8^+^ cell subsets and a more suppressive phenotype of T regulatory cells (Treg)^30^. Co-staining of LSP1 and TIM-3 revealed that CD4^+^ and CD8^+^ T cells expressing TIM-3 exhibited minimal or negligible LSP1 expression (Fig. 3B). This suggests that LSP1 is likely absent from the surface of exhausted CD4^+^ and CD8^+^ T cells.

### LSP1 expression is positively correlated with better survival and reflects the infiltration of tumour-associated immune cells in cervical cancer

**Fig. 4:**
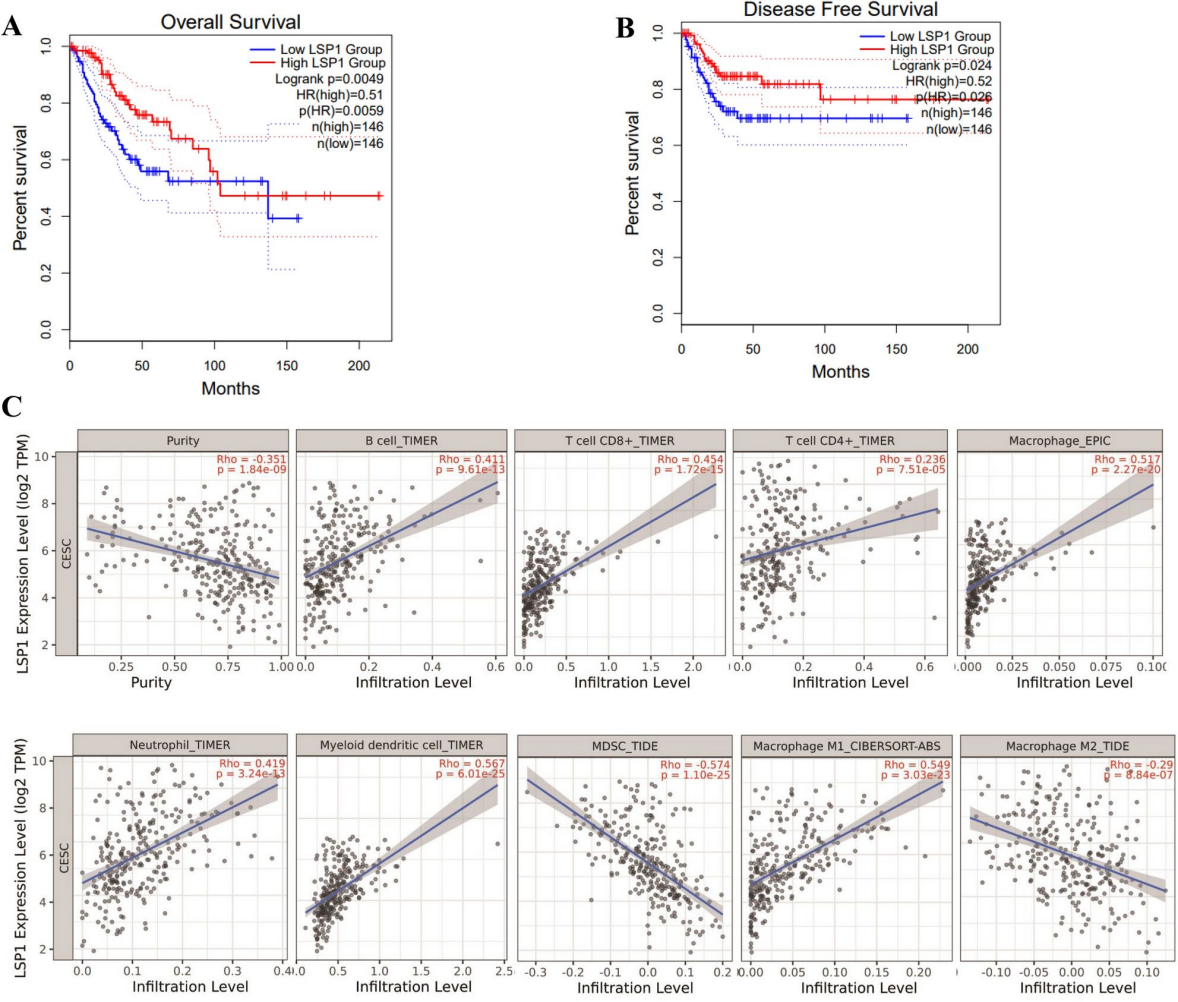
GEPIA survival analysis suggests that higher expression of LSP1 mRNA is associated with a better prognosis of cervical cancer patients, indicated by extended overall survival (OS, *p* = 0.0049, **A**) and disease-free survival (DFS, *p* = 0.024, **B**). (**C**) Scatter plots generated by TIMER2.0 analysis reveal that LSP1 transcript levels are negatively correlated with estimated tumour purity and tumour infiltration of myeloid-derived suppressor cells (MDSCs) and M2 macrophages, while positive correlations exist between LSP1 and the abundance of B cell, CD4^+^ T cell, CD8^+^ T cell, neutrophil, dendritic cell and M1 macrophage.

To determine the relationship between LSP1 gene expression and the prognosis of cervical squamous cell carcinoma (CESC) patients, the survival analysis module of the GEPIA database was analysed. We found that higher LSP1 expression prognosed a better overall survival (OS, HR = 0.51, *p* = 0.0049, Fig. 4A) and disease-free survival (DFS, HR = 0.52, *p* = 0.024, Fig. 4B) of the CESC patients. Based on the identification that LSP1 was negatively correlated with tumour purity, and positively correlated with the key immune infiltrates (Table 1), we used the TIMER2.0 and TISIDB databases to investigate the correlation between LSP1 expression and six main TILs (B cells, CD8^+^ T cells, CD4^+^ T cells, neutrophils, macrophages and dendritic cells) and immunomodulatory molecules including immunosuppressants, immunostimulants, MHC molecules and chemokines/receptors. The results demonstrated that LSP1 expression was negatively correlated with cervical squamous cell carcinoma (CESC) tumor purity (r =  − 0.351, p = 1.84e-09). Significant positive correlations were observed between LSP1 and the infiltration of B cells (r = 0.439, p = 8.96e-13), CD8^+^ T cells (r = 0.414, p = 8.96 e-13), CD4^+^ T cells (r = 0.453, p = 1.95e-15), and neutrophils (r = 0.513, p = 5.16e-20) (Fig. 4C). Additionally, a weak positive correlation was found between LSP1 and macrophage infiltration (r = 0.097, p = 1.06e-01). Using the TIMER2.0 database, we further analyzed the association between LSP1expression and specific immune cell types. The analysis revealed that LSP1 expression was positively correlated with the infiltration levels of M1 macrophages (Rho = 0.549, p = 3.03e-23) and dendritic cells (Rho = 0.604, p = 8.74e-29), but negatively correlated with M2 macrophages (Rho =  − 0.29, p = 8.84e-07) and myeloid-derived suppressor cells (Rho =  − 0.574, p = 1.10e-25) (Fig. 4C).

### LSP1 may be associated with immunostimulants, MHC molecules, chemokines, and chemokine receptors

**Fig. 5:**
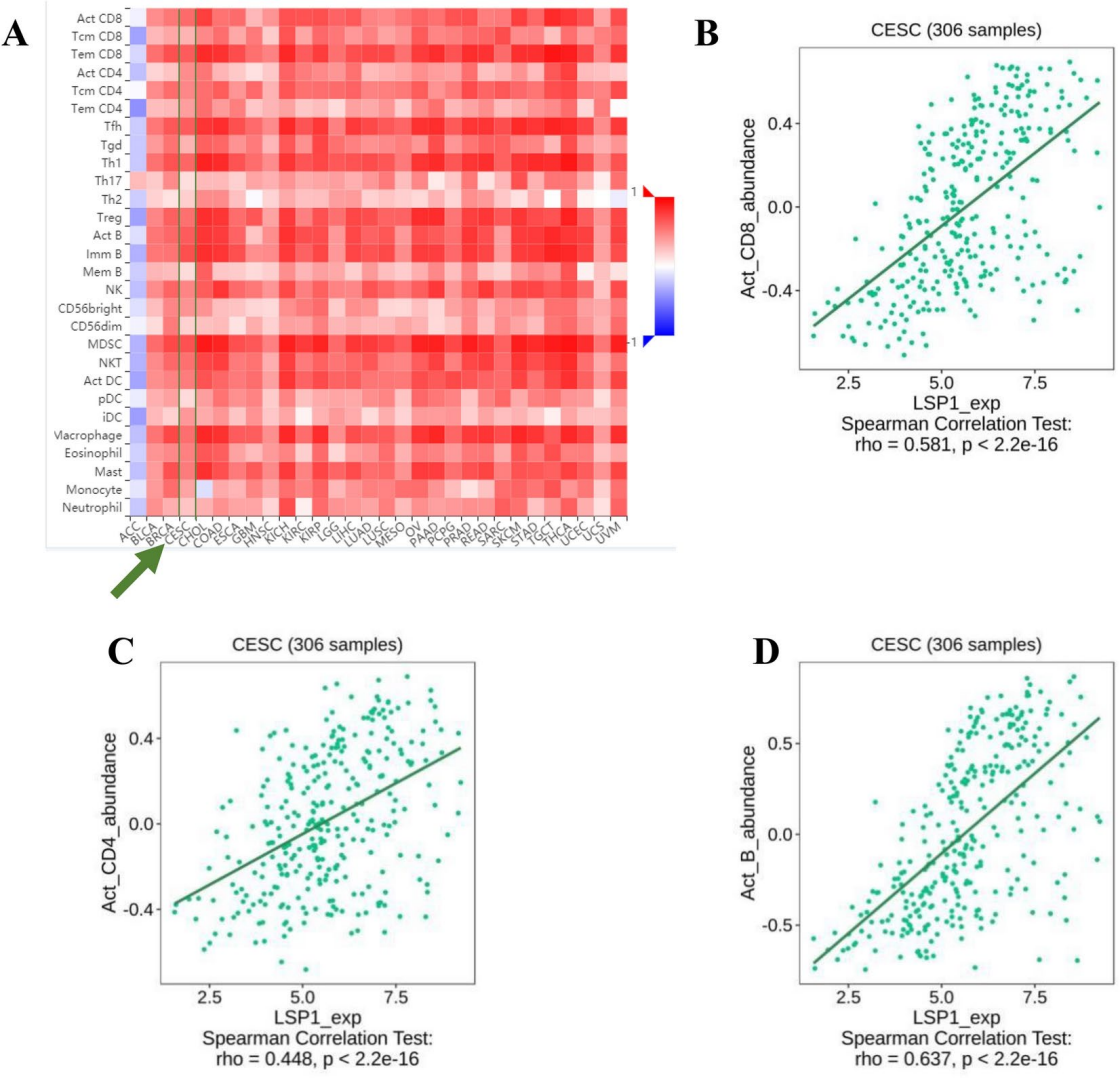
Heatmaps produced by TISIDB analysis showing LSP1 expression in association with TILs (**A**). The correlation between LSP1 and the abundance of activated CD8^+^ (**B**), effector memory and activated CD4^+^ T cell (**C**) and activated B cells (**D**) are highlighted.

Using the TISIDB database, we created a heatmap to analyse the detailed correlation between LSP1 and TILs in common tumours. Except for adrenocortical carcinoma (ACC), all types of solid tumours examined, such as bladder urothelial carcinoma (BLCA), breast invasive carcinoma (BRCA) and CESC **(**Fig. 5A**,** highlighted by green arrows and lines), exhibited positive correlations between LSP1 and infiltration of various tumour-associated lymphocytes. In particular, LSP1 expression was positively correlated with the abundance of activated CD4^+^ T cells, activated CD8^+^ T cells and activated B cells (Fig. 5B-D). The results were in accordance with our observations from mIHC,leading us to hypothesize that LSP1 may be expressed in non-exhausted CD8^+^ and CD4^+^ T cells (Fig. 3B).

**Fig. 6:**
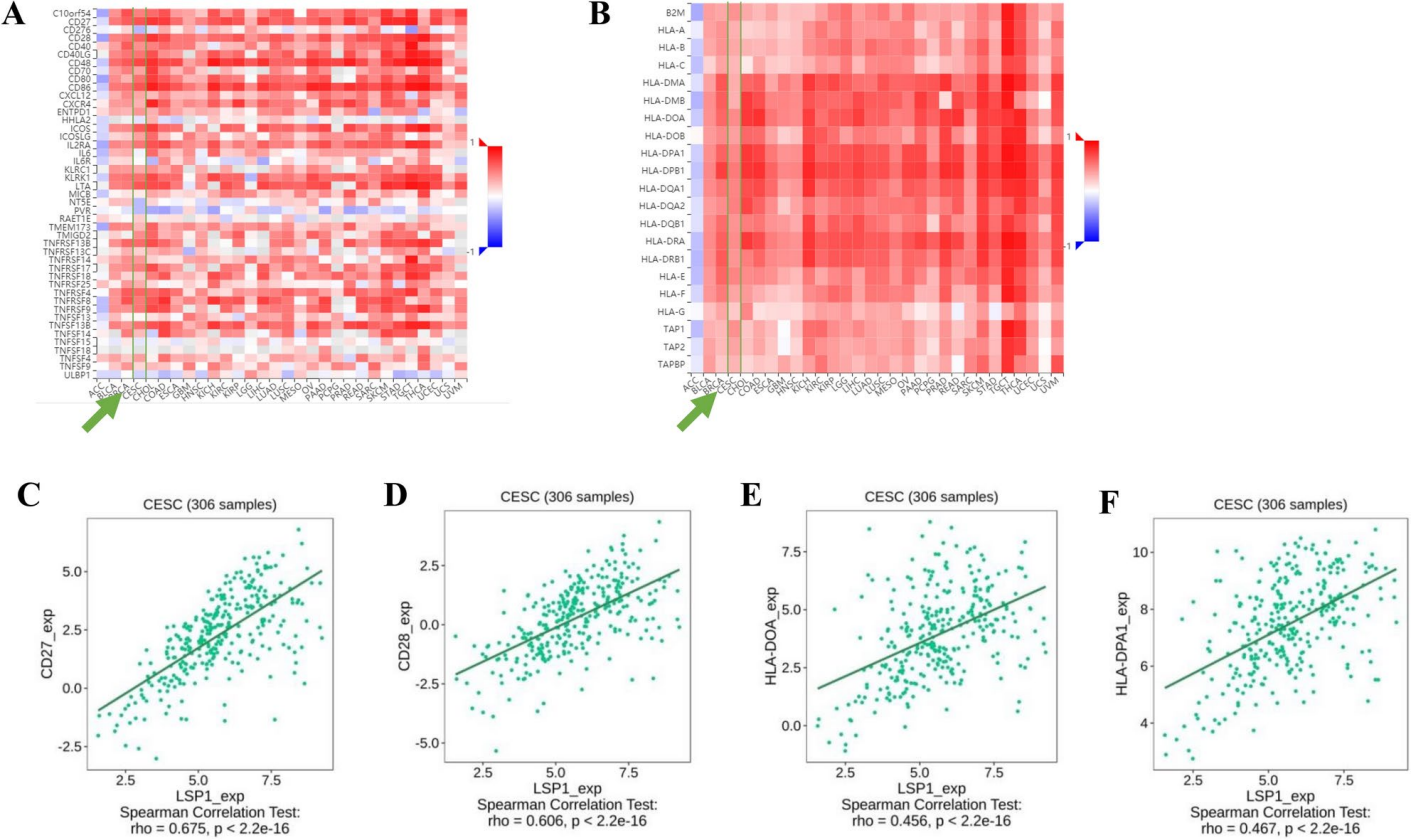
Heatmaps produced by TISIDB analysis showing LSP1 expression in association with immunostimulants (**A**) and MHC molecules (**B**). The correlations between LSP1 and the abundance of CD27 (**C**), CD28 (**B**)**,** HLA-DOA (**C**) and HLA-DPA1 (**D**) are highlighted.

Likewise, the correlation between LSP1 expression levels and immunostimulants **(**Figs. 6A) and MHC molecules (Figs. 6B) in common solid tumours were analysed. Costimulatory receptor molecules that mediate the signalling transduction pathways of T-cell proliferation, differentiation and activation, such as cluster of differentiation 27 (CD27) and 28 (CD28), have been proposed as important immunomodulation targets in cancer treatments^31,32^. We found that LSP1 expression was positively correlated with CD27 (Figs. 6C) and CD28 (Figs. 6D). Positive correlations between LSP1 levels and human leukocyte antigen (HLA) variants HLA-DOA (Fig. 6E**)** and HLA-DPA1 were also detected in CESC (Fig. 6F). Both HLA-DOA and HLA-DPA1 belong to class II major histocompatibility complex (MHC II) complexes, which have significant association with TME and clinical benefits including patient response to immunotherapy and cancer prognosis, as demonstrated by a pan-cancer association study^33^. The heatmap result supported that the beneficial upregulation of HLA-DPA1 positively correlated with high LSP1 levels (Fig. 6B**,** highlighted by green arrows and lines).

**Fig. 7:**
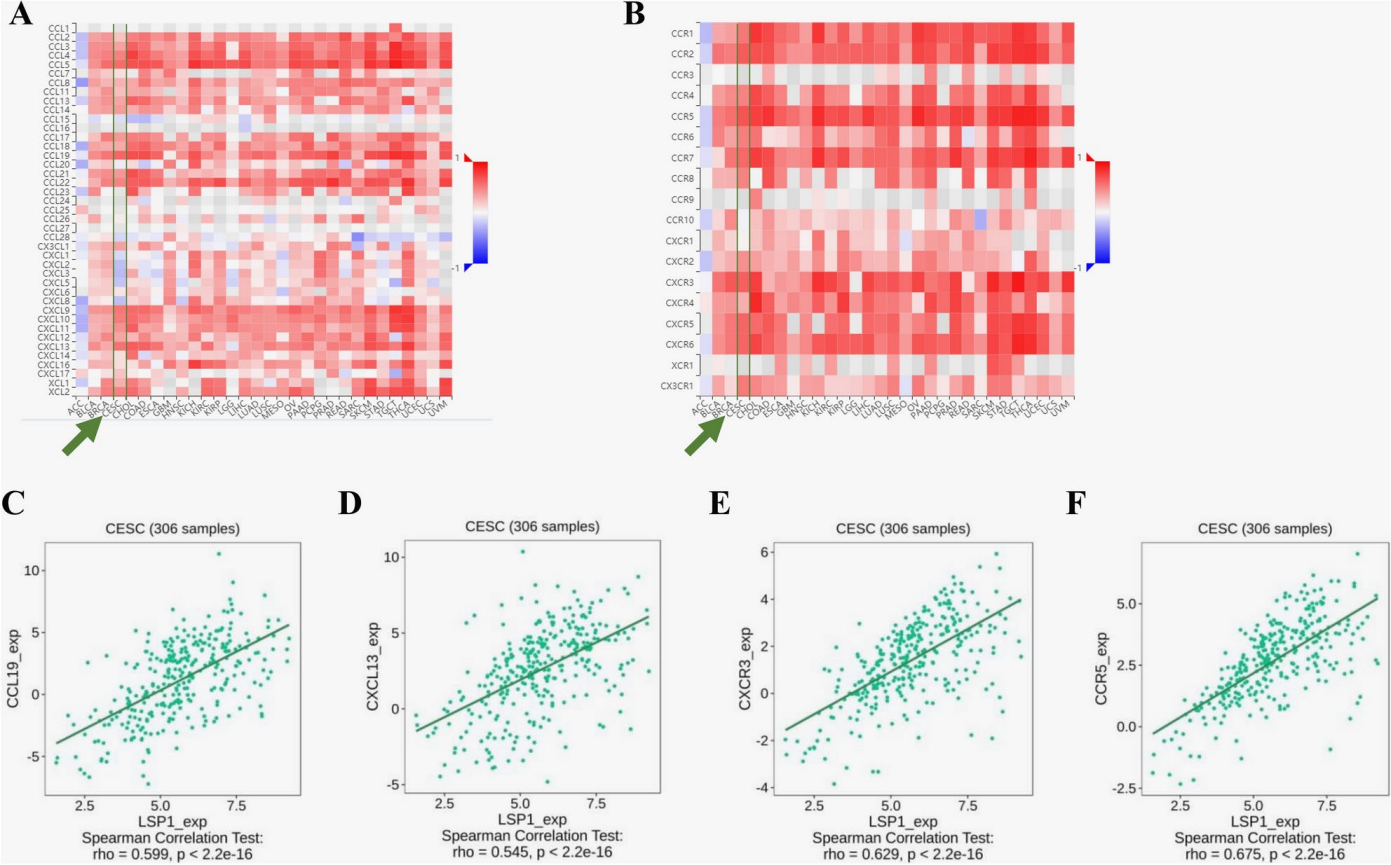
Heatmaps produced by TISIDB analysis showing LSP1 expression in association with immunochemokines (**A**) and chemokine receptors (**B**). The correlations between LSP1 and the abundance of CCL19 (**C**), CXCL13 (**D**), CXCR3 (**E**) and CCR5 (**F**) are highlighted.

In the immune niche of TME, the chemokines and chemokine receptors constitute essential signalling networks signalling the trafficking of immune cells. In tandem with other soluble TME components such as growth factors, these molecules also contribute to cancer-specific immunity, tumour growth and tumour angiogenesis. Various changes in several chemokines and chemokine receptors were detected by the heatmap analysis (Fig. 7A&B). LSP1 expression was positively correlated with CCL19, CXCL13, CXCR3 and CCR5 in CESC (Figs. 7C-F**)**. Although the changes in chemokines are consistent with previous findings in cervical cancer^34^, it would be premature to speculate the functional meanings of these correlations without further mechanistic investigations.

## Discussion

Over the course of cervical cancer initiation and progression, the immune landscape is constantly shaped and reshaped by the rivalry between HPV infection and host defence. The alterations of the subpopulations of immune cells and the composition of tumour-infiltrating lymphocytes (TILs) can lead to the loss of balance between immune activation and suppression within the tumour microenvironment (TME).

Our interest in LSP1 was piqued by its significant reduction observed in patient samples positive for HPV16 infection, irrespective of the presence of cervical cancer, as demonstrated by proteomics data. In non-cancerous tissues, the expression levels of LSP1 are relatively low, whereas in cancerous tissues, elevated LSP1 expression is associated with favorable immune infiltration.

The seemingly contradictory observations should not be too surprising. Although the identification of tumour-associated proteins from exfoliated cervical cells has been proven a powerful tool for cancer screening^35^, it has intrinsic limitations in the detection of aberrations in TME components they are not readily sloughed off and stay retained within the basal lamina of the tumour tissue. Moreover, using the HPLC-LIF technique, a previous study comparing normal and different stages of cervical malignancy has found significant variations in the protein profiles derived from tissue homogenate, exfoliated cervix cell lysate and serum samples^36^. Not only the stages of disease progression but also the sample types can have a drastic impact on the relative intensity, area under the curve and width of the individual protein peaks.

We detected that LSP1 was positively associated with both CD4^+^ and CT8^+^ T cell abundance in HPV16-positive cervical cancer. Infiltration of CD4^+^ or CD8^+^ T cells was found to signify a good prognosis for patients with cancers such as pancreatic ductal adenocarcinoma^37^ and lung adenocarcinoma^38^. However, the interpretation of infiltrating T-cell markers in cancer usually requires nuances of cancer stage and cell subtypes. A meta-analysis has been conducted to evaluate the associations between T-cell infiltrations and disease development and prognosis of cervical lesions and invasive cancers, in comparison to normal cervix. The study found that the infiltration of CD4^+^, assumably characterised as helper T-cells, was reduced in the benign side of the CIN spectrum but was the greatest in cancer, while the pattern of CD8 positivity that signifies active adaptive immunity was less consistent^39^.

A previous study reported^42^ that in HPV-positive cervical cancer, the expression level of LSP1 in the high-risk group is significantly upregulated compared to the low-risk group, which is contradictory to our research results. We hypothesize that LSP1 expression may be associated with the stage of cervical cancer. To investigate this, we collected clinical tissue samples and performed immunohistochemistry (IHC) analysis, revealing increased LSP1 expression in cervical cancer tissues relative to normal cervical tissues, particularly in Stage IB cervical cancer. Notably, LSP1 expression was lower in Stage IIIC cervical cancer tissues compared to Stage IB (Fig. 2C). Western blot and RT-qPCR analyses further confirmed these observations by demonstrating consistent protein and mRNA expression levels of LSP1 in early and advanced cervical cancer tissues. Survival analysis using the GEPIA database indicated that high LSP1 expression correlates with improved patient prognosis. Previous studies have shown that in head and neck squamous cell carcinoma^40^, CD8^+^ T cell infiltration increases from stage I to III, but their function is inhibited by the PD-L1/PD-1 immune checkpoint. In osteosarcoma patients^41^, TIM-3 expression on peripheral CD4^+^ and CD8^+^ T cells positively correlates with tumor stage, with advanced-stage patients exhibiting higher TIM-3 expression. High expression of TIM-3^+^ CD4^+^ Th1 cells and TIM-3^+^ CD8^+^ T cells plays tumour-promoting roles in HPV-positive cervical cancer^29^. Elevated TIM-3 expression in TME marks the exhaustion of CD8^+^ cell subsets and a more suppressive phenotype of T regulatory cells (Treg)^30^. Our multiple immunohistochemistry (mIHC) data demonstrate that LSP1 is predominantly expressed on CD8^+^ and CD4^+^ T cells with low TIM-3 expression. This suggests that the reduced LSP1 expression in advanced-stage tumors may be associated with an exhausted T cell phenotype or a more pronounced inhibitory phenotype of regulatory T cells (Tregs) within the tumor microenvironment (TME).

Meanwhile, we found that the presence of LSP1 expression was negatively correlated with immune suppressive cells such as M2 macrophages and MDSCs which are supposed to encourage cervical carcinogenesis by maintaining an immunosuppressive TME, promoting angiogenesis and metastasis, and impairing CD8^+^ cytotoxicity and antigen presentation^43^. The role of LSP1 in regulating cytoskeletal architecture, cell motility and chemotaxis of immune cells, and activation of class II MHC is critical in inflammatory responses^44^. Besides, by breaking the cellular symmetry, LSP1 interacts with actin and other myosin activity regulators to enable the polarization and migration of immune cells, especially macrophages where LSP1 is a podosome component^45^. In conjunction with existing knowledge, our results suggest that LSP1 might be tumour-suppressive in cervical cancer. Future studies will further investigate the mechanisms and clinical significance of LSP1 in the activation of anti-tumor immunity in cervical cancer.

The elimination of virus-infected or tumoral cells generally relies on cytotoxic CD8^+^ T cells, whose effects depend on the recognition of peptide-MHC complexes^46^. We found that the expression level of LSP1 was significantly positively correlated with the expression level of MHC-II molecules HLA-DOA and HLA-DPA1 and predicted better patient survival. This agrees with studies in which the MHC-II signature was proposed as an independent and favourable predictor of immune response and progress of bladder cancer under treatment with immune checkpoint inhibitors^47^. While lack of MHC-I presentation is often among the main causes of tumour immune escape, MHC-II molecules are strongly associated with effector T-cells and immune activation^47^.

Therapeutic blockade of immune checkpoint underpins current immune therapy strategies, by using inhibitors to break the immune tolerance and enhance anti-tumour activity. TIM-3 is one of the known immune checkpoint receptors that inhibit the signal transduction of T-cell activation, thereby inhibiting effector T-cell functions. As TIM-3 expression marks the terminal differentiation and exhaustion of T-cells and is often co-expressed with other important checkpoint inhibitors such as PD-1 in cancer, TIM-3 expression on CD4^+^ and CD8^+^ T cells is considered a cardinal sign of T-cell dysfunctional^30^. The mutual exclusion of LSP1 and TIM-3 expression on CD4^+^ and CD8^+^ T cells in the cervical cancer tissues examined in this study inferred that LSP1 and TIM-3 may exhibit counteracting effects on immune cells in TME. The benefits of TIM-3 inhibition have been demonstrated in experiments on a TIM-3-deficient mouse model of colon carcinoma. Deletion of TIM-3 on CD4^+^ and CD8^+^ T cells reduced tumour burden and loss of TIM-3 in DC cells enhanced antigen-specific anti-tumour immunity, expanded stem-like CD8^+^ cells and activation inflammasome pathways^48^.

In different disease contexts, LSP1 is known to modulate the migration of T-cells^26^, B-cells, neutrophils^49,50^ and macrophages^45^. In the present study, LSP1 was found significantly positively correlated with the expression levels of CCL19, CXCL13, CXCR3 and CCR5, suggesting the role of LSP1 in the migration of immune cells into the TME. As a downstream regulatory molecule of CXCL12/CXCR4 signal transduction, low LSP1 expression results in a significant increase of B-cell chemotaxis^51^. As mentioned in the earlier part of the paper, opposite observations were reported in studies of different cancers, such as in a mouse model of B16 melanoma, LSP1 knockout improved T-cell infiltration and cytotoxicity^26^. CD8^+^ T cells from melanoma tumour mass of LSP1 KO mice exhibited higher migration ability in response to CXCL9 and CXCL10, whereas T cell-specific LSP1 overexpression in transgenic mice resulted in suppressed immune infiltration and promoted melanoma growth^26^.

In conclusion, inflicted by persistent HPV16 invasion, the microenvironment of the cervical tissue undergoes significant changes that entail dynamic immune cell trafficking, which is significantly associated with LSP1 expression. Notwithstanding the potential role of LSP1 as a tumour suppressor in cervical cancer, we are not able to claim any causal link of LSP1 expression to anti-tumour immunity in cervical cancer. We also could not answer the question of why LSP1 protein abundance in the exfoliated cell lysates was significantly lower in samples with HPV16 infection. We could not rule out the possibility that LSP1 might be a surrogate signature of the TME perturbations. To definitively conclude whether LSP1 could be a potential prognostic marker or therapeutic target for cervical cancer treatment, cell-based mechanistic studies and functional assessment of TILs in clinical samples are needed.

## Conclusion

The expression level of LSP1 in cervical tissue increases in the early stages of cervical cancer (CC), decreases with disease progression, and is positively associated with a favorable prognosis. The expression of LSP1 exhibits a positive correlation with the abundance of major TILs and immune regulatory molecules, particularly activated B cells, CD8^+^ T cells, and CD4^+^ T cells. Conversely, LSP1 demonstrates a negative correlation with the abundance of M2 macrophages and MDSCs. This study suggests that LSP1 may serve as a valuable biomarker for determining the stage and prognosis of cervical cancer and is likely involved in the infiltration and activation of anti-tumor immune cells within the TME. The potential value of LSP1 as a novel target for staging and treatment stratification in cervical cancer warrants further investigation.

## Materials and methods

### Clinical samples and study design

As described in our previous study in which the same patient cohort was examined^27^, samples of cervical exfoliated cells were collected in the Affiliated Hospital of Guizhou Medical University and the Affiliated Cancer Hospital of Guizhou Medical University from December 2017 to July 2018. Subjects with any of the following medical histories were excluded: smoking, alcohol consumption, hypertension, diabetes, coronary heart disease, other tumours, radiotherapy, chemotherapy or other drug therapy. A total of 135 samples were collected and divided into three groups with an equal number of subjects (n = 45): HPV-negative normal group (group A, aged 43.44 ± 7.78), HPV16-positive normal group (group B, aged 42.44 ± 10.68), and HPV16-positive cervical cancer (squamous cell carcinoma) group (group C, aged 45.80 ± 8.5). There were no significant inter-group differences in age (F = 1.623, *p*-value = 0.201). The diagnosis of cervical squamous cell carcinoma was made according to the standards of Expert Consensus on Cervical Cancer Screening and Abnormal Management in China, in conjunction with the medical history, cytological examination and pathological biopsy results. The normal group referred to women with no abnormal cervical cytology. HPV DNA test was conducted on cervical exfoliated cells using Real-time fluorescence PCR reverse spot hybridization method, following standard genotyping protocol. Three biological replicates were performed in each group, which were A1, A2, and A3; B1, B2, and B3; C1, C2, and C3. Each replicate was generated by mixing up 15 randomly chosen samples of exfoliated cervical cells. All replicates within the groups were age-matched. Subgroups of A (F1 = 0.686, *p*-value = 0.509) were: A1 (44.67 ± 7.55), A2 (44.13 ± 5.64) and A3 (41.83 ± 9.7). Subgroups of B (F2 = 0.026, *p*-value = 0.974) were: B1 (42.60 ± 11.97), B2 (41.93 ± 10.64) and B3 (42.80 ± 10.09); Subgroups of C (F3 = 0.008, *p-*value = 0.992) were: C1 (45.80 ± 10.03), C2 (46.00 ± 8.86) and C3 (45.6 ± 6.91). Pathological sections of formalin-fixed paraffin-embedded (FFPE) hysterectomy specimens were collected from patients with confirmed pathological diagnosis of HPV16-positive cervical cancer (cancer group, n = 20) or HPV16-negative benign hysteromyoma (normal group, n = 20), provided by the Department of Pathology, Affiliated Hospital of Guizhou Medical University for IHC verification. 2 samples out from the cancer group were selected for mIHC verification.According to the FIGO staging of cervical cancer, additional cervical cancer tissue specimens were collected from patients who attended the Department of Gynecology of Guizhou Medical University Hospital from September 2020 to September 2024 with surgical excision and paraffin-embedded cervical cancer tissues, including normal cervical tissues (n = 4), early stage of cervical cancer (Stage IB) tissues (n = 6), and advanced stage of cervical cancer (Stage IIIC) tissues (n = 4),Normal cervical tissues were obtained from patients who underwent hysterectomy for leiomyoma or adenomyoma; surgically excised cervical cancer tissue specimens from patients attending the Department of Gynecology of Guizhou Medical University Hospital were also collected, and they were divided into cancerous and paracancerous groups.All samples collected and tested have been approved by the Ethics Committee of the Affiliated Hospital of Guizhou Medical University and informed consent was obtained from all participants.

### Tandem mass tags (TMT)-labelled quantitative proteomics

Our previous study^27^ identified differentially expressed proteins (DEPs) through liquid chromatography-mass spectrometry/mass spectrometry (LC–MS/MS) analysis. Briefly, 100 μg proteins were extracted and digested from cellular samples in each group and labelled with TMT before loading into high-performance liquid chromatography fractionation (HPLC) and liquid chromatography-mass spectrometry/mass spectrometry (LC–MS/MS). DEPs were identified and quantified using Proteome Discoverer TM 2.2 (Thermo Fisher, USA) and UniProt human database(https://www.uniprot.org/). Fold change (FC) > 2 or FC < 0.5 was defined as significantly up-regulated or down-regulated, respectively. Student’s *t*-test *p*-value < 0.05 was used as the threshold for screening the proteins with significant differences. Cluster analyses were plotted by R scripts (ComplexHeatmap2.4.3).

### TIMER & TISIDB analyses

The TCGA data-based web server Tumour Immune Estimation Resource (TIMER 2.0, http://timer.cistrome.org/) was exploited to screen differentially expressed common proteins associated with tumour immune infiltration^52^. Spearman’s correlation coefficients were used to evaluate the correlation between DEPs, tumour purity and the abundances of immune infiltrates of CD8^+^ T cell, CD4^+^ T cell, B cell, macrophage, neutrophil, and dendritic cell in cervical cancer. *p*-value < 0.05 was considered statistically significant.

The web portal TISIDB (http://cis.hku.hk/TISIDB), designed for crosschecking tumour-immune interactions^53^ was then used to analyze the correlations between LSP1 expression and different immune characteristics including tumour-infiltrating lymphocytes (TILs), immunomodulators (immunostimulators, immune inhibitors, and major histocompatibility complex molecules), chemokines and chemokine receptors. Spearman’s correlation coefficients were computed and *p*-value < 0.05 was considered statistically significant.

### GEPIA (gene expression profiling interactive analysis) survival analysis

The web tool GEPIA based on TCGA and GTEx data for multi-functional expression analyses was used (http://gepia.cancer-pku.cn/)^54^. By choosing the “Survival Analysis” module, the prediction performance of LSP1 for “Overall Survival (OS)” and “Disease-Free Survival (DFS)” in CESC (Cervical squamous cell carcinoma) patients were investigated. The group cut-off was set as “Median”.

### Immunohistochemistry (IHC)

For IHC, 3-μm sections of FFPE cervical tissue were prepared. Sections underwent dewaxing, antigen retrieval, and blocking of endogenous peroxidase activity using a 3% hydrogen peroxide solution. The sections were then blocked with 10% goat serum for 30 min at room temperature. Primary antibodies against LSP1 (rabbit, 1:200, Abcam), CD4 (rabbit, 1:500, Abcam), and CD8 (rabbit, 1:800, Abcam) were applied for incubation. Following incubation with secondary antibodies (goat anti-rabbit IgG, 1:5000, Zhongshanjinqiao, China), chromogen 3,3’-diaminobenzidine (DAB; ZLI-9018, Zhongshanjinqiao, China) was used to produce brown staining in positive areas. Sections were subsequently dehydrated, cleared, mounted, and examined under a Nikon ECLIPSE 80i microscope. Hematoxylin was used for counterstaining. The pictures were analyzed by Image J.

### Western blot analysis

The tissues were washed and subsequently homogenized in radioimmunoprecipitation assay (RIPA) lysis buffer (Solarbio, China). Protein concentrations were determined using a bicinchoninic acid (BCA) Protein Assay Kit (Solarbio, China). Proteins were then separated via 10% sodium dodecyl sulfate–polyacrylamide gel electrophoresis (SDS-PAGE) and transferred onto a 0.45 μm polyvinylidene difluoride (PVDF) membrane (Merck Millipore, Tullagreen, Ireland). The membranes were blocked with 5% skim milk, followed by incubation with primary and secondary antibodies. Detection was performed using an electrochemiluminescence (ECL) system (BIO-RAD ChemiDoc™ XRS^+^, USA). The primary antibodies used were Anti-LSP1 (Ab133506, 1:20,000; Abcam, UK) and Anti-GAPDH (ET1601-4, 1:50,000; HuaBio, China). The secondary antibody was biotinylated goat anti-rabbit immunoglobulin G (BS13278, 1:8,000; Bioworld, China).

### Real-time quantitative PCR analysis

Total cellular RNA was extracted using the TRIzol® Reagent. Samples were transferred to RNase-free Eppendorf tubes and incubated at room temperature for 5 min. Chloroform (200 µl) was added, followed by gentle inversion for 15 s and incubation at room temperature for 3 min to facilitate phase separation. Subsequently, samples were centrifuged at 12,000 × g for 15 min at 4 ℃. The aqueous phase was carefully collected and transferred to new RNase-free Eppendorf tubes, after which 400 µl of isopropanol was added. Further RNA purification was conducted according to the protocol provided with the Stratec RNA isolation kit. RNA quality was assessed based on the A260/A280 ratio (~ 2.0) using a NanoDrop™ spectrophotometer (Thermo Fisher Scientific). Reverse transcription of RNA into cDNA was performed using the PrimeScript™ RT Reagent Kit with gDNA Eraser (TaKaRa, Japan), following the manufacturer’s instructions. Quantitative real-time PCR (qRT-PCR) was carried out on an Applied Biosystems 7500 Real-Time PCR System (Thermo Fisher Scientific) using SYBR Green master mix. GAPDH served as the reference gene. Amplification conditions consisted of 45 cycles, each comprising denaturation at 95℃ for 30 s, annealing at 55℃ for 30 s, and extension at 72℃ for 20 s. Relative RNA expression levels were quantified using the 2 − ΔΔCt method.

LSP1:5’-AGG ACC GAG TCC CTA AAC CG-3’ (forward) and 5’-CTG GGT GTA TTG CAG CCA-3’(reverse);GAPDH: 5’- GGA GCG AGA TCC CTC CAA AAT-3’ (forward) and 5’- GGC TGT TGT CAT ACT TCT CAT GG-3’ (forward).

### Multiplex immunohistochemistry(mIHC)

mIHC staining was performed on the histopathologic sections of cervical cancer to detect the distribution of CD4^+^ and CD8^+^ T cells around blood vessels and their co-expressions with LSP1. Following the heating–cooling cycle for antigen retrieval and blocking with 10% goat serum, the sections were simultaneously labelled with primary antibodies (Abcam) against CD4 (1:600), CD8 (1:800), CD31 (1ug/ml), LSP1 (1:100) and TIM-3 (1:500). After incubation with suitable secondary antibodies (Absin) and fluorescent immunostaining with tyramide signal amplification (TSA, Absin, ab50012), an automated Vectra 3.0 quantitative pathology imaging system was utilized for visualization.

### Statistical analysis

Statistical analysis was performed with the Statistical Program for Social Sciences (SPSS) (SPSSInc., version 22.0, United States). The quantitative data were reported as the means ± standard deviation (SD), and the significant difference was analysed with a *t*-test between two groups, a single factor analysis of variance for comparison of three groups. *p*-value < 0.05 was considered statistically significant.

## Supplementary Information

Supplementary Information 1.

Supplementary Information 2.

## Data Availability

The data used to support the findings of this study are available from the corresponding author upon request.
