# Supplementary material for: Leukocyte-specific protein 1 is associated with the stage and tumor immune infiltration of cervical cancer
Source: Sci Rep. 2025 Mar 4;15:7566. doi: 10.1038/s41598-025-91066-0 (PMC11880245; doi:10.1038/s41598-025-91066-0)
Supplement: Supplementary file 2 — Supplementary Information 2. [file 41598_2025_91066_MOESM2_ESM.pdf]

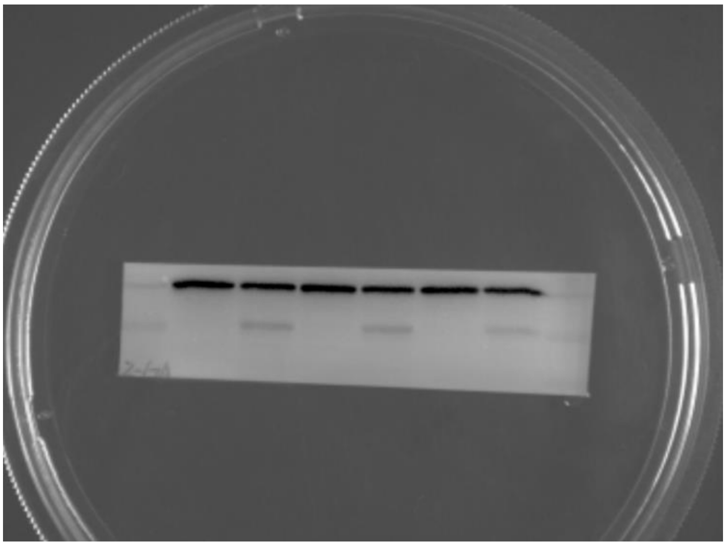

Stage Ⅰ B GAPDH（P1C1P2C2P3C3）

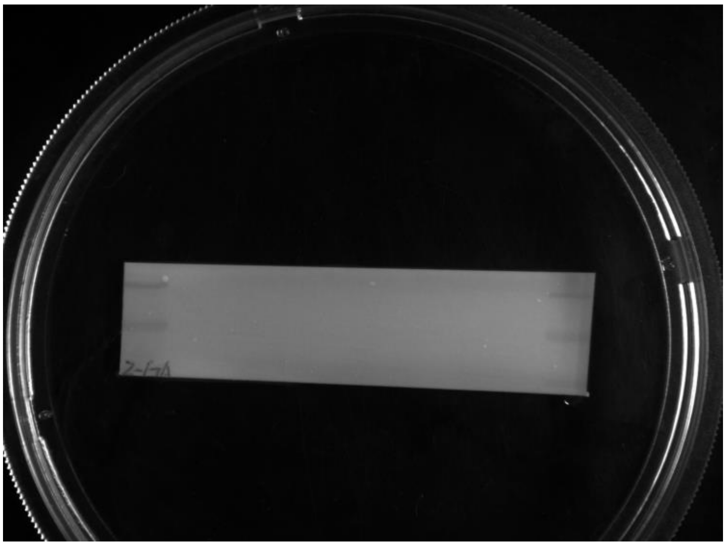

Stage Ⅰ B GAPDH（P1C1P2C2P3C3）Marker

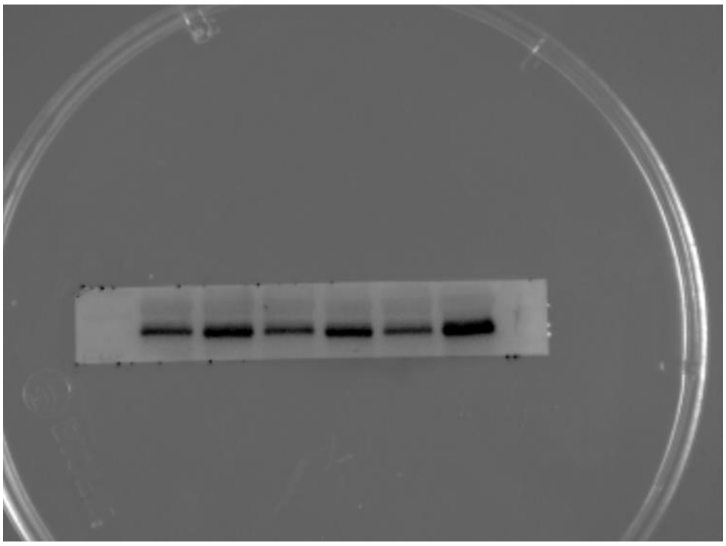

Stage Ⅰ B LSP1（P1C1P2C2P3C3）

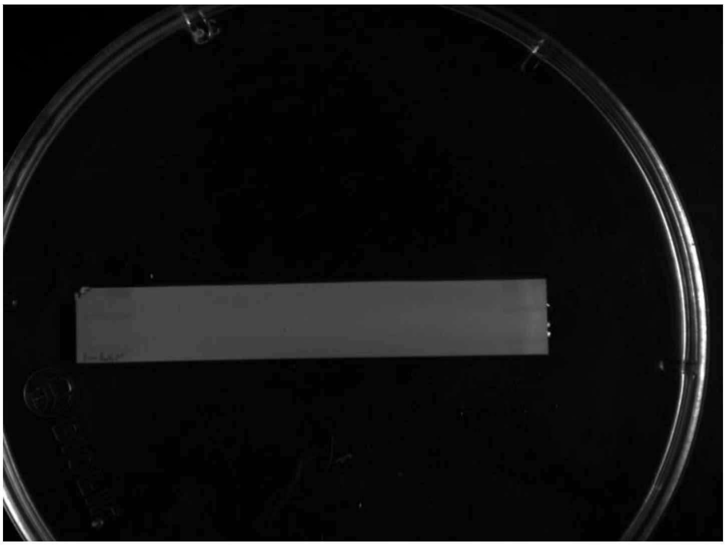

Stage ⅠB LSP1（P1C1P2C2P3C3）Marker

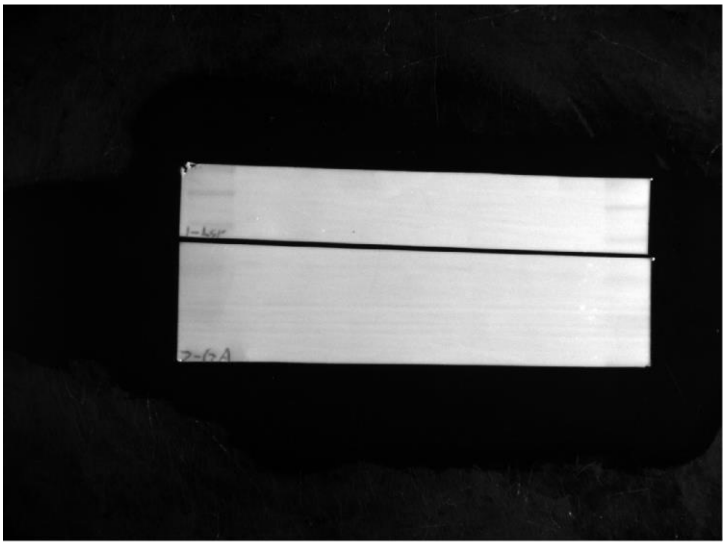

Stage ⅠB

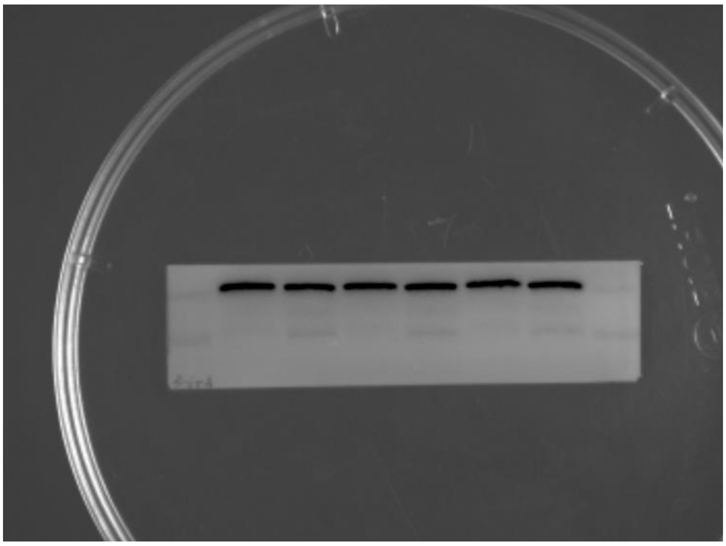

Stage ⅢC GAPDH（P4C4P5C5P6C6）

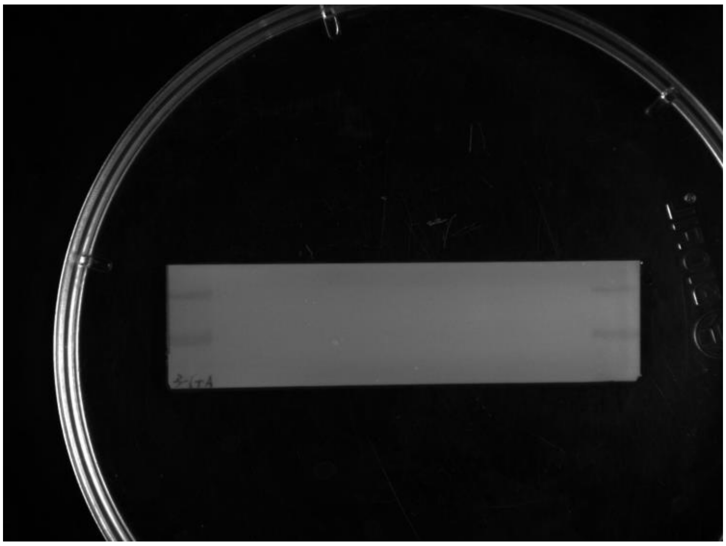

Stage ⅢC GAPDH（P4C4P5C5P6C6）Marker

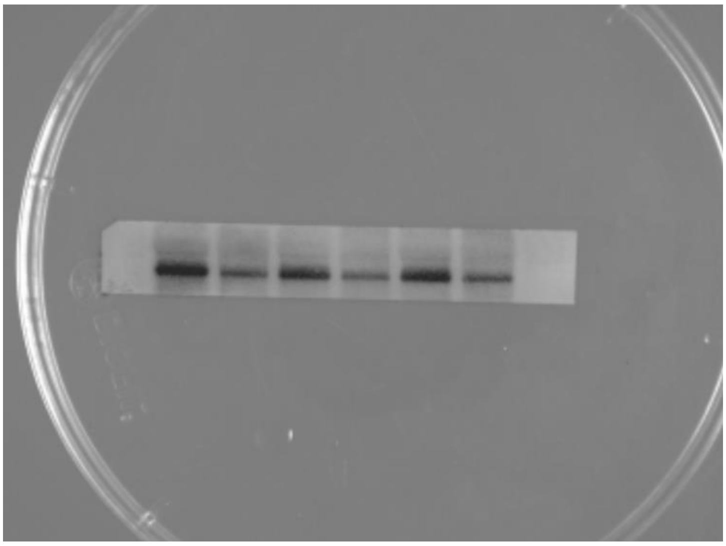

Stage ⅢC LSP1（P4C4P5C5P6C6）

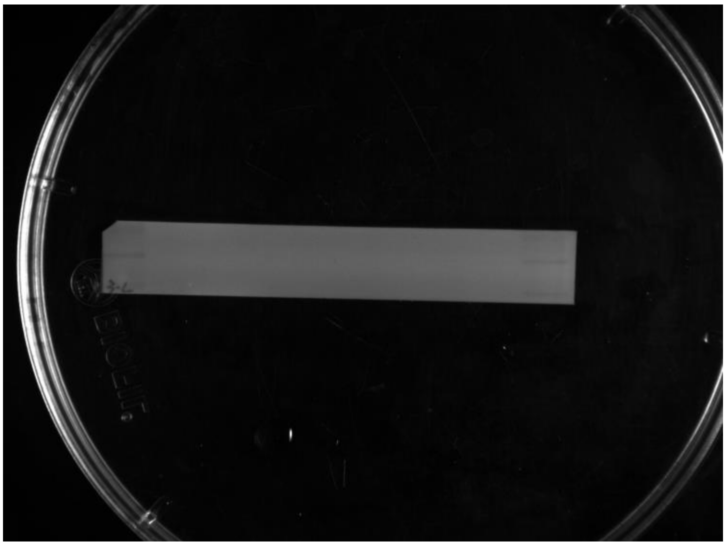

Stage ⅢC LSP1（P4C4P5C5P6C6）Marker

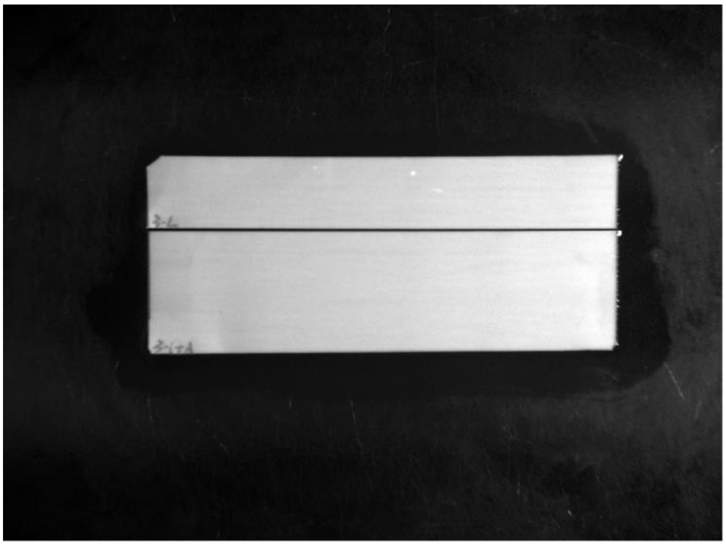

Stage ⅢC
